# Deep learning based automatic internal gross target volume delineation from 4D‐CT of hepatocellular carcinoma patients

**DOI:** 10.1002/acm2.14211

**Published:** 2023-11-22

**Authors:** Zhen Yang, Xiaoyu Yang, Ying Cao, Qigang Shao, Du Tang, Zhao Peng, Shuanhu Di, Yuqian Zhao, Shuzhou Li

**Affiliations:** ^1^ Department of Oncology National Clinical Research Center for Geriatric Disorders Xiangya Hospital Central South University Changsha China; ^2^ School of Automation Central South University Changsha China

**Keywords:** 4D‐CT, automated delineation, deep learning, hepatocellular carcinoma, internal gross target volume

## Abstract

**Background:**

The location and morphology of the liver are significantly affected by respiratory motion. Therefore, delineating the gross target volume (GTV) based on 4D medical images is more accurate than regular 3D‐CT with contrast. However, the 4D method is also more time‐consuming and laborious. This study proposes a deep learning (DL) framework based on 4D‐CT that can achieve automatic delineation of internal GTV.

**Methods:**

The proposed network consists of two encoding paths, one for feature extraction of adjacent slices (spatial slices) in a specific 3D‐CT sequence, and one for feature extraction of slices at the same location in three adjacent phase 3D‐CT sequences (temporal slices), a feature fusion module based on an attention mechanism was proposed for fusing the temporal and spatial features. Twenty‐six patients’ 4D‐CT, each consisting of 10 respiratory phases, were used as the dataset. The Hausdorff distance (HD95), Dice similarity coefficient (DSC), and volume difference (VD) between the manual and predicted tumor contour were computed to evaluate the model's segmentation accuracy.

**Results:**

The predicted GTVs and IGTVs were compared quantitatively and visually with the ground truth. For the test dataset, the proposed method achieved a mean DSC of 0.869 ± 0.089 and an HD95 of 5.14 ± 3.34 mm for all GTVs, with under‐segmented GTVs on some CT slices being compensated by GTVs on other slices, resulting in better agreement between the predicted IGTVs and the ground truth, with a mean DSC of 0.882 ± 0.085 and an HD95 of 4.88 ± 2.84 mm. The best GTV results were generally observed at the end‐inspiration stage.

**Conclusions:**

Our proposed DL framework for tumor segmentation on 4D‐CT datasets shows promise for fully automated delineation in the future. The promising results of this work provide impetus for its integration into the 4DCT treatment planning workflow to improve hepatocellular carcinoma radiotherapy.

## 1 INTRODUCTION

Radiotherapy is one of the primary treatment options for hepatocellular carcinoma (HCC). Preoperative radiotherapy helps shrink the tumors and prepare the patient for surgery,^1^, ^2^ and postoperative radiotherapy helps consolidate the curative effect and im‐prove the prognosis of patients,^3^, ^4^ so that this dual approach has become the clinical consensus for standard of care. For this to be effective the precise delineation of the tumor target volume is essential. However, the liver's location and morphology are significantly affected by respiratory motion, making the necessary level of precision difficult to obtain. Previous studies have shown that liver displacement due to respiratory motion can reach as much as 0.5‐5.0 cm.^5^ Adverse effects caused by respiratory motion during radiotherapy planning and delivery can also include the following: the presence of artifacts in planned CT scans, leading to the inaccurate delineation of tumor and normal tissue con‐tours; altered dosimetry based on static planning; and increased irradiated volume of normal tissue,^6^, ^7^, ^8^ especially for intensity‐modulated radiotherapy. To address this issue, the International Commission on Radiation Units and Measurements (ICRU) introduced the concept of Internal Target Volume (ITV) in its No. 62 report.^9^ The ITV consists of an internal margin added to the Clinical Target Volume (CTV) to compensate for internal physiologic movement and variations in size, shape, and position of the CTV. Currently, there is no commonly accepted definition of ITV area, however. The ITV does not need to be delineated when designing the radiotherapy plan, but the tumor motion must be accounted for when delineating the Planning Target Volume (PTV). Some studies have thus derived the concept of Internal Gross Tumor Volume (IGTV), which explicitly accounts for internal variations in tumor location, size, and shape, and is also macroscopically visible.^10^

4DCT helps us assess motion of the organs at simulation, and alter the way we plan and deliver radiation accordingly. Specifically, 4D‐CT incorporates a temporal dimension to conventional 3D CT, enabling improved temporal resolution while maintaining the same intensity and spatial resolution. 4D‐CT uses helical scan mode to acquire images, and in clinical practice, 4D‐CT scans typically divide the respiratory cycle into 8–10 phases, each corresponding to a complete set of 3D‐CT images. The tumor's size, shape, and location on CT can thus be individualized for each phase. Since 4D‐CT contains respiratory motion information of the target volume, IGTV formed by GTV extrapolation based on patient‐specific target motion characteristics allows for the determination of individualized IGTV and reduces the risk of target under coverage or over‐irradiation of normal tissues.^11^, ^12^

The value of 4D‐CT in precise radiotherapy for liver cancer has been generally acknowledged as well. Theoretically, the ideal IGTV delineation method based on 4D‐CT is achieved by acquiring images of each respiratory phase and delineating the GTV separately then fusing them all together. However, this dramatically increases the workload of radiation oncologists. The current solution to this problem is to use commercially available deformable image registration methods for contour propagation, although the generated contours of such methods have large uncertainties, according to Gianfranco et al.,^13^ the DSC of thoracic ITV was 0.74 ± 0.11, and abdominal CTV was 0.7 ± 0.05, which is not yet clinically available. Therefore, designing an effective computer‐aided method is essential to facilitating automatic GTV delineation in all respiratory phases.

Recently, deep learning (DL) has been successfully applied to medical image analysis, including automatic registration, segmentation, and dose prediction, and has been shown to achieve state‐of‐the‐art performance. Several algorithm competitions about liver tumor segmentation are also currently ongoing; various advanced algorithms are continually tested and improved on publicly available MRI and CT datasets. The most noteworthy of which are Dircadb,^14^ LiTS,^15^ Silver07,^16^ and Chaos,^17^ but only a few studies have proposed DL frameworks based on 4D‐CT datasets and demonstrated the feasibility of automatically localizing and segmenting tumors directly on 4D‐CT datasets.

Similar studies to this one includes that of the motion convolutional neural networks (R‐CNN) framework proposed by S Momin et al., which was used to segment lung tumors on 4D‐CT image sets rapidly and accurately.^18^ Additionally, Zheng et al. proposed a DL model based on 3D convolution and C‐LSTM that utilized 4D information from multi‐phase DCE images for automatic liver tumor segmentation.^19^ In fact, existing DL seg‐mentation algorithms perform well enough on 3D CT image data. We tested U‐Net with the LITS dataset, and for 2D U‐Net, a DSC of 0.946 ± 0.085 can be obtained for liver segmentation. In this work, we propose a new spatial‐temporal dual‐path framework for segmenting liver tumors in 4D‐CT datasets by exploiting convolutional neural networks' robust feature extraction capabilities. The network consists of two encoding paths, one for feature extraction of adjacent slices (spatial slices) in a specific 3D‐CT sequence, and one for feature extraction of slices at the same location in three adjacent phase 3D‐CT sequences (temporal slices). In addition, we propose a feature fusion module based on an attention mechanism for fusing the temporal and spatial features. To measure the effectiveness of this approach, we compare these results to the network without the fusion module and to manually delineated labels.

## 2 MATERIALS AND METHODS

### 2.1 Algorithm flow

**FIGURE 1:**
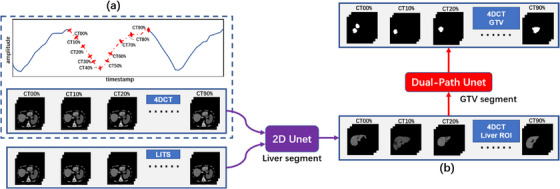
The workflow of the proposed method. A small part of the acquired 4D‐CT is combined with the LITS dataset as the training dataset for the liver segmentation model. After obtaining the prediction results for liver ROI, the GTV is segmented within the ROI using the dual‐path U‐Net, and the dataset used for the GTV segmentation model is 4D‐CT.

Figure 1 presents the flow of our method. The segmentation process consists of two independent stages, first segmenting the liver with U‐Net, then segmenting the GTV with the proposed Dual‐path network on liver ROI, this scheme can reduce the region to be detected by the model and improve the segmentation efficiency. CT00% to CT90% in Figure 1a are the reconstructed 3D‐CTs corresponding to each phase of the breathing cycle, which belong to the same 4D‐CT set with the identical settings for scanning and reconstruction, so there is no significant difference in quality (spatial resolution, density resolution or noise) between them. Since the acquired 4D‐CTs are not used as planning CTs, there are generally no OARS or GTVs on them, and the labels used for the training are lacking. In addition, delineating the liver on all 4D‐CT datasets is time‐consuming and costly in terms of labor. To solve this problem, we referred to the transfer learning method to obtain better segmentation results by delineating the liver labels on only a small number of 4D‐CTs. Specifically, our radiation oncologists delineated the liver on ten 4D‐CT sets. They then resized the 4D‐CTs and liver labels to be consistent with the LITS dataset by cropping and resampling (the LITS is a liver tumor segmentation benchmark whose data are provided by various clinical sites. The training data set contains 130 CT scans and the test data set 70 CT scans^15^), and six 4D‐CT sets were thusly added to the LITS training dataset. Then the dataset was preprocessed with a unified procedure as the training dataset for liver segmentation, which significantly increased the sample size for training and improved the model's prediction accuracy.

### 2.2 Spatial‐temporal dual path U‐Net architecture

**FIGURE 2:**
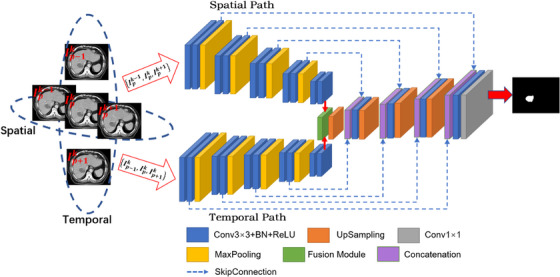
The schematic representation of the dual‐path network structure. The structure consists of a temporal path and a spatial path each with three adjacent temporal slices and three adjacent spatial slices as inputs. The bottom layer of the encoding path is the feature fusion module, which is used to fuse the temporal and spatial features.

The proposed network is presented in Figure 2. The encoding part of the network consists of spatial paths and temporal paths. A 4D‐CT can be represented as a four‐dimensional tensor $I\in R^{H\times W\times L\times P}$, where H, W, and L denote the resolution of the anterior‐posterior, left‐right, and superior‐inferior directions of the patient, respectively, and P denotes the temporal phase number. The 3D‐CT of the P temporal phase can then be expressed as a 3D tensor $I_{p}\in R^{H\times W\times L}$ itself consisting of L slices, where the *k^th^* slice can be expressed as $I_{p}^{k}$, 0≤k≤L. The two slices adjacent to the *k^th^* slice in the superior and inferior directions can be expressed as $I_{p}^{k+1}$ and $I_{p}^{k+1}$, and the two slices adjacent to the $I_{p}^{k}$ slice in phase can be denoted as $I_{p+1}^{k}$ and $I_{p-1}^{k}$. The input of the spatial path of the network is $I^{S}=\left\{I_{p}^{k-1},I_{p}^{k},I_{p}^{k+1}\right\}$, and the input of the temporal path is $I^{T}=\left\{I_{p-1}^{k},I_{p}^{k},I_{p+1}^{k}\right\}\;$.

Both spatial and temporal encoding paths are composed of five encoding layers. The 3D‐CT of different phases can thus be interpreted as the sampling and reconstruction results of different time intervals within the same respiratory cycle of the patient, which contains temporal information, so the two paths of the network are designed to be parallel, with the same structure for their encoding layers. Each encoding layer contains two consecutive convolutional modules and a pooling module. The purpose of using stacked convolutional layers like this is to increase the depth of the network. As the feature maps propagate through the encoding paths, the receptive field of the network gradually expands, enabling the extraction of more profound and abstract spatio‐temporal information. Batch Normalization (BN) and the Rectified Linear Unit (ReLU) function are introduced in the module to connect the convolutional layers, adjust the model parameters, retain the valuable features, and discard the features with little relevance to the segmentation target. Maximum pooling is used for downsampling at the end of each encoding module to reduce the feature map resolution, which is adjusted to $\frac{H}{16}\times \frac{W}{16}$ after four downsamplings. The temporal and spatial features are fully extracted in the parallel dual encoding path and will then be input to the feature fusion module to establish associations.

### 2.3 Fusion module

**FIGURE 3:**
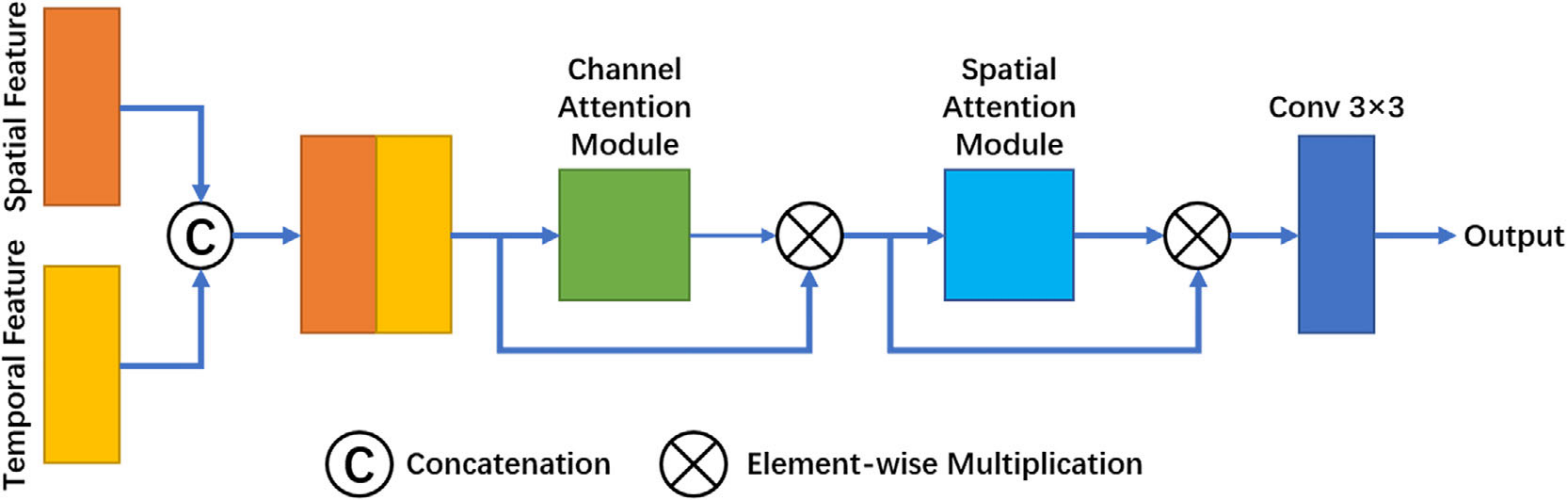
Feature fusion module. The module consists of a channel attention module and a spatial attention module. First, the feature maps from the bottom layers of the two encoding paths are concatenated then sequentially passed through the two attention modules to establish the connection of spatial‐temporal features. Finally, the number of channels is adjusted by the convolution layer and input into the decoding path.

Inspired by CBAM (Convolutional Block Attention Module), we designed our spatial‐temporal feature fusion module to have a channel attention module and a spatial attention module, as shown in Figure 3. First, the spatial‐temporal feature maps from the bottom layer of the encoding path are concatenated. Since each channel of the feature map can be regarded as a feature detector, the channel attention mechanism focuses on the useful information of the inputs. The feature map is then dimensionally compressed in the spatial direction by global average pooling and maximum pooling to refine and aggregate spatial and temporal information, and then passed to the shared multilayer perceptron (MLP) component. Finally, the feature vectors are merged, and the channel attention module is formulated as:

(1) $$\begin{array}{ccc} M_{C}\left(\left[F_{s},F_{t}\right]\right) & = & \sigma \left(MLP\left(AP\left(\left[F_{s},F_{t}\right]\right)\right)\right.\\ & & +\;\left.MLP\left(MP\left(\left[F_{s},F_{t}\right]\right)\right)\right) \end{array}$$

where $M_{C}$ denotes the Channel Attention Module, σ denotes the Sigmoid function, brackets denote Concatenation, AP denotes Average Pooling, and MP denotes Max Pooling.

A new feature map $F^{\prime }\in R^{C\times H\times W}$ is obtained after adjustment by the channel attention mechanism and then passed to the spatial attention module, which focuses attention on where the temporal and spatial information comes from. As a complement to the channel attention module, the temporal and spatial information within the feature map is then refined and fused. Global average pooling and maximum pooling are performed along the channel direction, and the obtained feature maps are concatenated to generate effective feature representations, which are then convolved to generate the spatial attention map $M_{S}\left(F^{\prime }\right)\in R^{H\times W}$. The spatial attention module is formulated as:

(2) $$M_{S}\left(F^{\prime }\right)=\sigma \left(\;\;\sum_{i}\sum_{j}k\left(i,j\right)*\left(\left[AP\left(F^{\prime }\right),\;MP\left(F^{\prime }\right)\right]\right)\;\;\right)$$

where $M_{S}$ denotes the Spatial Attention Module, $\sum_{i}\sum_{j}k\left(i,j\right)*$ denotes Convolution, the convolution kernel size is *i* = *j* = 7, and the number of channels is adjusted to 1 by Convolution. The feature maps output by the channel attention module and spatial attention module are element‐wise multiplied, yielding the final feature map $F^{\prime \prime }\in R^{C\times H\times W}$. Thus, feature correlation between the spatial and temporal paths is established, the attention weight associated with spatial‐temporal features is enhanced, and the weightings of redundant and invalid information are reduced.

### 2.4 Loss function

Liver tumor segmentation is a class imbalance issue. In order to segment the tumor effectively, we implemented the BCEWithLogitsLoss function to decrease the imbalance between the numbers of tumor and background pixels. This function combines the Sigmoid layer and the BCELoss function, making it more numerically stable. The BCEWithLogitsLoss function is defined as follows:

(3) $$\begin{array}{ccc} l\left(x,y\right) & = & L={\left\{l_{1},\text{…},l_{N}\right\}}^{T},\\ l_{n} & = & \;-w_{n}\left[y_{n}\cdot log_{x_{n}}+\left(1-y_{n}\right)\cdot log\left(1-x_{n}\right)\right] \end{array}$$

(4) $$l\;\left(x,y\right)=\;mean\left(L\right)$$

where N is the batch size.

### 2.5 Metrics

The metrics we used for quantitative comparison were the Dice Similarity Coefficient (DSC), the 95% Hausdorff Distance (HD95), and the Volume Difference (VD). The formulas for each are expressed as follows:

(5) $$DSC=2\frac{\left|A\cap B\right|}{\left|A\right|+\left|B\right|}$$

(6) $$\begin{array}{c} \;HD_{95}\;=\;max\left\{\max_{x\in X}\min_{y\in Y}d_{95}\left(x,y\right),\;\max_{y\in Y}\min_{x\in X}d_{95}\left(x,y\right)\right\} \end{array}$$

(7) $$VD\;=\left|Volume_{GT}-Volume_{prediction}\right|$$

where A denotes the predicted result, B denotes the ground truth, and d_95_ is the HD95 (Tables 1 and 3).

### 2.6 4D‐CT dataset

The planned and imaging data of 26 patients with HCC who received radiotherapy at our institution were selected for this study, patient characteristics and the mean respiration‐induced liver motion are presented in Table 2. All 4D‐CTs were acquired using the Siemens SOMATOM Definition AS CT scanner with the following acquisition parameters: tube voltage of 140 kV, tube current of 150 mA, image reconstruction with a slice thickness of 3 mm, pixel size of 0.977 mm, and a matrix of 512 × 512. All 4D‐CT images were acquired with the patient breathing freely, and each patient was briefly trained to breathe as smoothly and evenly as possible before the scan. We used a laser‐based optical surface scanning system (C‐RAD Sentinel 4D‐CT) for 4D‐CT reconstruction. The 4D software in Sentinel sorted the data of all positions and respiratory phases once the scan was completed and reconstructed 10 sets of 3D‐CTs that contained the information of each respiratory phase (0−90%), as well as the Maximum Intensity Projection (MIP). Here, 0% represents the end‐inspiratory phase, 50% represents the end‐expiratory phase, and the other remaining phases are all intermediate respiratory states. A typical respiratory curve is given in Figure 1a, where the vertical axis represents respiratory amplitude, and the horizontal axis represents timestamps. The respiratory curve amplitude in the figure did not show significant fluctuations, and the frequency was stable, meeting the criteria for 4D‐CT reconstruction. After the reconstruction was completed, all images were imported to the Eclipse treatment planning system (Varian Medical Systems, Palo Alto, California, USA) for GTV delineation.

**TABLE 1:** Network parameters of dual‐path U‐Net.

Spatial path			Temporal path		
Layer name	Kernel size	Output size	Layer name	Kernel size	Output size
Input		512×512×1	Input		512×512×1
Conv_1	3×3	512×512×64	Conv_1	3×3	512×512×64
Conv_1_1	3×3	512×512×64	Conv_1_1	3×3	512×512×64
Conv_2	3×3	256×256×128	Conv_2	3×3	256×256×128
Conv_2_1	3×3	256×256×128	Conv_2_1	3×3	256×256×128
Conv_3	3×3	128×128×256	Conv_3	3×3	128×128×256
Conv_3_1	3×3	128×128×256	Conv_3_1	3×3	128×128×256
Conv_4	3×3	64×64×512	Conv_4	3×3	64×64×512
Conv_4_1	3×3	64×64×512	Conv_4_1	3×3	64×64×512
Conv_5	3×3	32×32×1024	Conv_5	3×3	32×32×1024
Conv_5_1	3×3	32×32×1024	Conv_5_1	3×3	32×32×1024
Fuse		32×32×1024			
Conv_6	3×3	64×64×1024			
Conv_7	3×3	128×128×512			
Conv_8	3×3	256×256×256			
Conv_9	3×3	512×512×128			
Conv_10	3×3	512×512×1			

**TABLE 2:** Patient characteristics and amplitudes of respiration‐induced liver motion.

Number	Gender	Age	Number of lesions	Tumor size (cm^3^)	location	Abdominal compression	Amplitude (mm)	
							AP	SI
1	M	46	2	403.7	RL	Yes	2.1	6.6
2	M	45	2	47.1	RL	No	7.5	12.1
3	F	33	1	20.1	LL	Yes	5.1	9.7
4	F	39	1	71.3	RL	No	7.6	14.3
5	M	65	1	133.8	RL	Yes	5.6	12
6	F	58	4	124.9	RL	Yes	4.3	12.7
7	M	52	1	134.5	RL	Yes	5.3	6.5
8	M	59	1	70.0	RL	No	7.1	16.1
9	M	65	1	50.6	RL	Yes	7.6	13.3
10	M	36	2	33.4	LL	Yes	3.8	5.4
11	M	45	1	39.1	RL	No	7.9	14.7
12	M	80	1	75.2	RL	No	6.5	14.8
13	F	31	1	30.5	RL	Yes	7.7	8.8
14	M	55	1	206.9	RL	No	6.8	11.6
15	M	34	2	51.9	LL	No	5.7	8.9
16	M	41	2	55.6	RL	Yes	5.3	10.4
17	M	79	1	11.9	RL	Yes	4.8	10.6
18	M	67	1	13.3	LL	No	8.0	11.4
19	F	55	1	22.4	LL	Yes	5.7	11.9
20	M	42	1	99.2	RL	No	7.2	8.7
21	F	56	2	59.8	RL	Yes	4.5	6.9
22	M	61	1	34.3	RL	Yes	7.8	9.3
23	M	54	2	61.2	LL	No	6.6	9.7
24	F	44	1	27.3	RL	Yes	4.5	5.3
25	F	74	1	56.1	RL	Yes	4.1	6.2
26	M	52	1	71.6	RL	Yes	5.9	10.6

Abbreviations: AP, Anterior‐Posterior; LL, Left Lobe; RL, Right Lobe; SI , Superior‐Inferior.

**TABLE 3:** Quantitative results of liver segmentation.

Dataset	Model	DSC	HD95 (mm)
LITS	2D U‐Net	0.946 ± 0.085	3.98 ± 2.83
LITS + 4D‐CT	2D U‐Net	0.971 ± 0.076	3.66 ± 2.61

The segmentation model employed is 2D U‐Net, and the data sets are LITS and LITS+4D‐CT.

The object of study and discussion in this paper is GTV. We had two experienced radiation oncologists manually delineate the GTV contours on all 10 sets of 3D‐CTs of each patient and then fused the GTVs of all respiratory phases to form the IGTV contours, which were used as the ground truth for comparison of the automatic delineation results of the DL model. Thus, we obtained 260 complete sets of 3D‐CTs from 26 patients’ 4D‐CTs, with corresponding GTV labels, which was sufficient for training the medical image segmentation model. We use 180 sets of these CTs as the training set, 30 as the validation set, and 50 as the test set.

## 3 RESULTS

**FIGURE 4:**
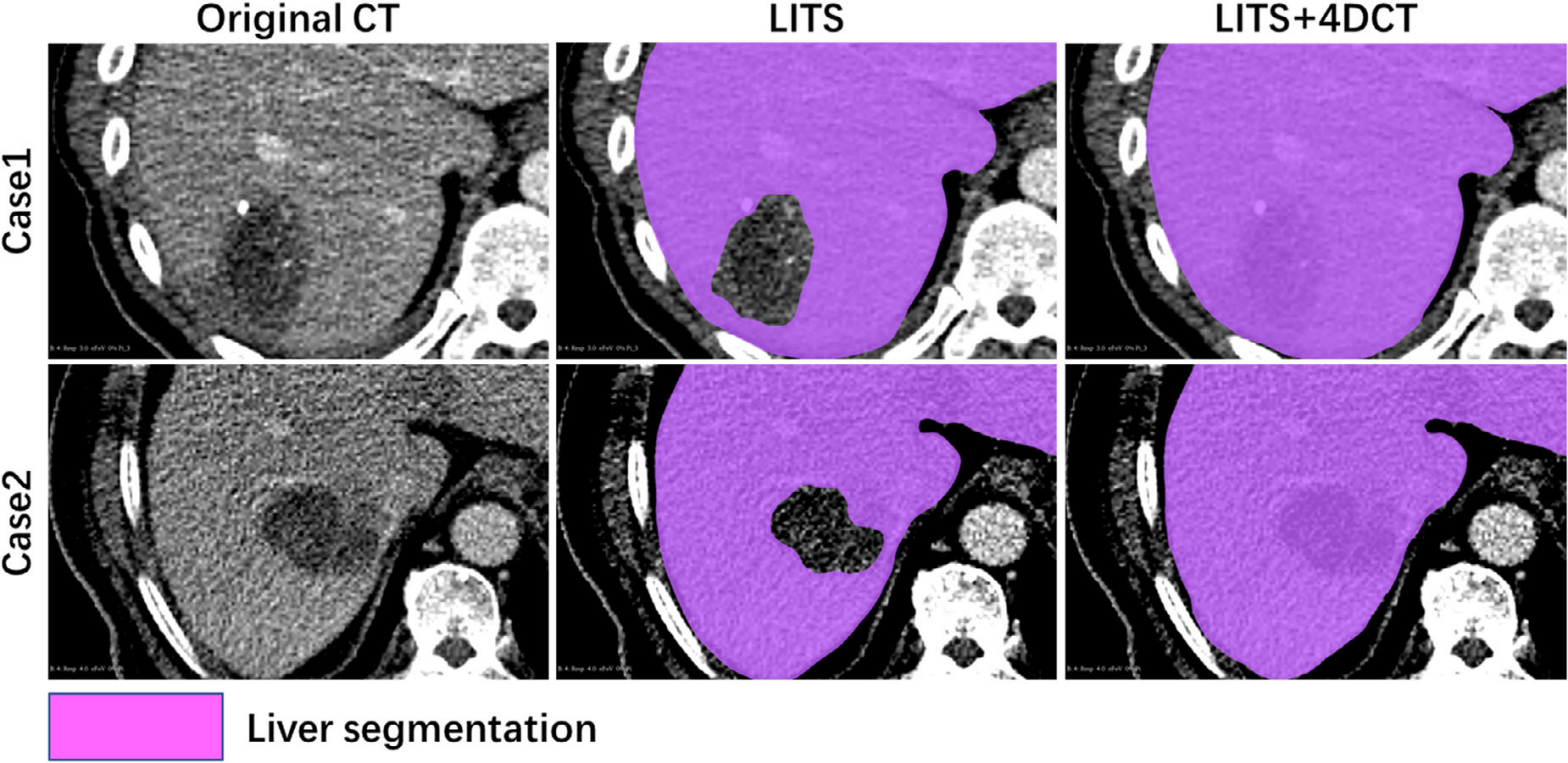
Visualization of liver segmentation results. The prediction model using only LITS as the training dataset identified the tumor region but incorrectly concluded that this region did not belong to the liver. The model with the LITS + 4D‐CT dataset did not have this problem.

In order to conduct visual comparison and quantitative analysis with the ground truth, we reassembled the predicted contours of each respiratory phase slice by slice, then converted them to Dicom format and set the 3D image size and centroid position to be the same as the corresponding ground truth. All visualization and evaluation matrix calculations in this paper were done in 3D Slicer software. Figure 4 shows the results of liver segmentation after post‐processing. When predicting the test set of 4D‐CT, the model trained with only the LITS dataset had an average DSC of 0.946 for the liver. Due to the high contrast between tumor and liver tissue of 4D‐CT, the model misclassified tumor as not part of the liver ROI, with a null region in the contour, as shown in the middle part of Figure 4. The prediction model obtained by adding the 4D‐CT data set to the training set significantly alleviated this misclassification, however, and improved the average DSC of the liver to 0.971. We carefully checked the 4D‐CT test set and found that the liver contour had ultimately been repaired, and the null area generated by the misclassification of the model was eliminated.

**FIGURE 5:**
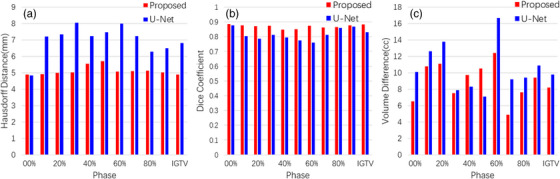
The results of the algorithm performance evaluation in terms of HD95, DSC, and VD for two methods applied for automated internal gross target volume delineation from 4D‐CT.

We used U‐Net as the base model to verify the effectiveness of our proposed method. The input of U‐Net was adjusted to $I\;=\{I_{p}^{k-1},I_{p-1}^{k},I_{p}^{k},I_{p+1}^{k},I_{p}^{k+1}\}\;$ to eliminate the effect caused by the inconsistent number of images of the model input, and the quantitative results for the tumor are presented in Table 4 and Figure 5. Here we see that our method outperformed U‐Net in all metrics. The best results were generally observed in the end‐inspiratory phases (00%, 10%, 80%, 90%), where the best performance of all evaluation indices was achieved in the 00% phase, with a DSC of 0.888 and an HD95 index of 4.90 mm. The end‐expiratory phases (40%, 50%) performed slightly poorer compared to the end‐inspiratory phase, with the 40% phase presenting the poorest results, with a DSC of 0.848, while the 50% phase, which is also the end‐inspiratory phase, presented the highest HD95 of 5.7 mm.

**TABLE 4:** A summary of the automatic delineation results, with the best results generally appearing at the end‐inspiratory phase (00%, 90%) and relatively poor results at the end‐expiratory phase (40%, 50%). The metrics for the IGTV are promising. We used a paired *t*‐test to compare the metric results of our proposed method with those of U‐Net, revealing statistically significant differences. This indicates a significant improvement in segmentation accuracy with our proposed method.

Method	Phase	HD_95_ (mm)	DSC	VD (cc)
Proposed	GTV00%	4.9 ± 2.33	0.888 ± 0.072	6.5 ± 5.2
	GTV10%	4.91 ± 2.21	0.878 ± 0.076	10.8 ± 8.3
	GTV20%	4.98 ± 2.59	0.87 ± 0.083	11.1 ± 9.6
	GTV30%	5.03 ± 3.37	0.875 ± 0.079	7.5 ± 5.4
	GTV40%	5.55 ± 3.76	0.848 ± 0.093	9.7 ± 8.7
	GTV50%	5.7 ± 3.82	0.851 ± 0.102	10.5 ± 9.5
	GTV60%	5.06 ± 3.45	0.873 ± 0.089	12.4 ± 10.2
	GTV70%	5.11 ± 3.38	0.861 ± 0.084	4.9 ± 5.2
	GTV80%	5.12 ± 3.63	0.864 ± 0.097	7.6 ± 6.9
	GTV90%	5.01 ± 2.96	0.878 ± 0.072	9.4 ± 6.1
	IGTV_all‐phases_	4.88 ± 2.84	0.882 ± 0.085	8.2 ± 6.9
U‐Net + 5slices	GTV00%	4.84 ± 3.18	0.876 ± 0.089	10.1 ± 8.9
	GTV10%	7.2 ± 4.95	0.804 ± 0.097	12.6 ± 11.3
	GTV20%	7.34 ± 6.22	0.785 ± 0.108	13.8 ± 10.8
	GTV30%	8.06 ± 5.86	0.813 ± 0.087	7.9 ± 7.3
	GTV40%	7.23 ± 4.71	0.794 ± 0.11	8.3 ± 7.9
	GTV50%	7.47 ± 5.08	0.773 ± 0.102	7.1 ± 7.6
	GTV60%	8 ± 6.13	0.761 ± 0.091	16.7 ± 12.8
	GTV70%	7.24 ± 5.27	0.812 ± 0.093	9.2 ± 9.4
	GTV80%	6.29 ± 4.92	0.858 ± 0.105	9.4 ± 8.9
	GTV90%	6.5 ± 4.63	0.868 ± 0.113	10.9 ± 10.1
	IGTV_all‐phases_	6.82 ± 4.87	0.829 ± 0.096	9.8 ± 9.2
	*p*	<0.01	<0.01	<0.01

We also compared the volumes of the GTV for each phase with the ground truth and found that our results were generally over‐segmented, with the average predicted GTV volume for all patients being reported as 9.04 cc larger than the ground truth. In contrast, the IGTV formed by merging the predicted GTV of all phases demonstrated improved results over the above, with an average over‐segmented volume of only 8.2cc compared to the ground truth. The DSC and HD95 also presented better results of 0.882 and 4.88 mm, respectively.

**FIGURE 6:**
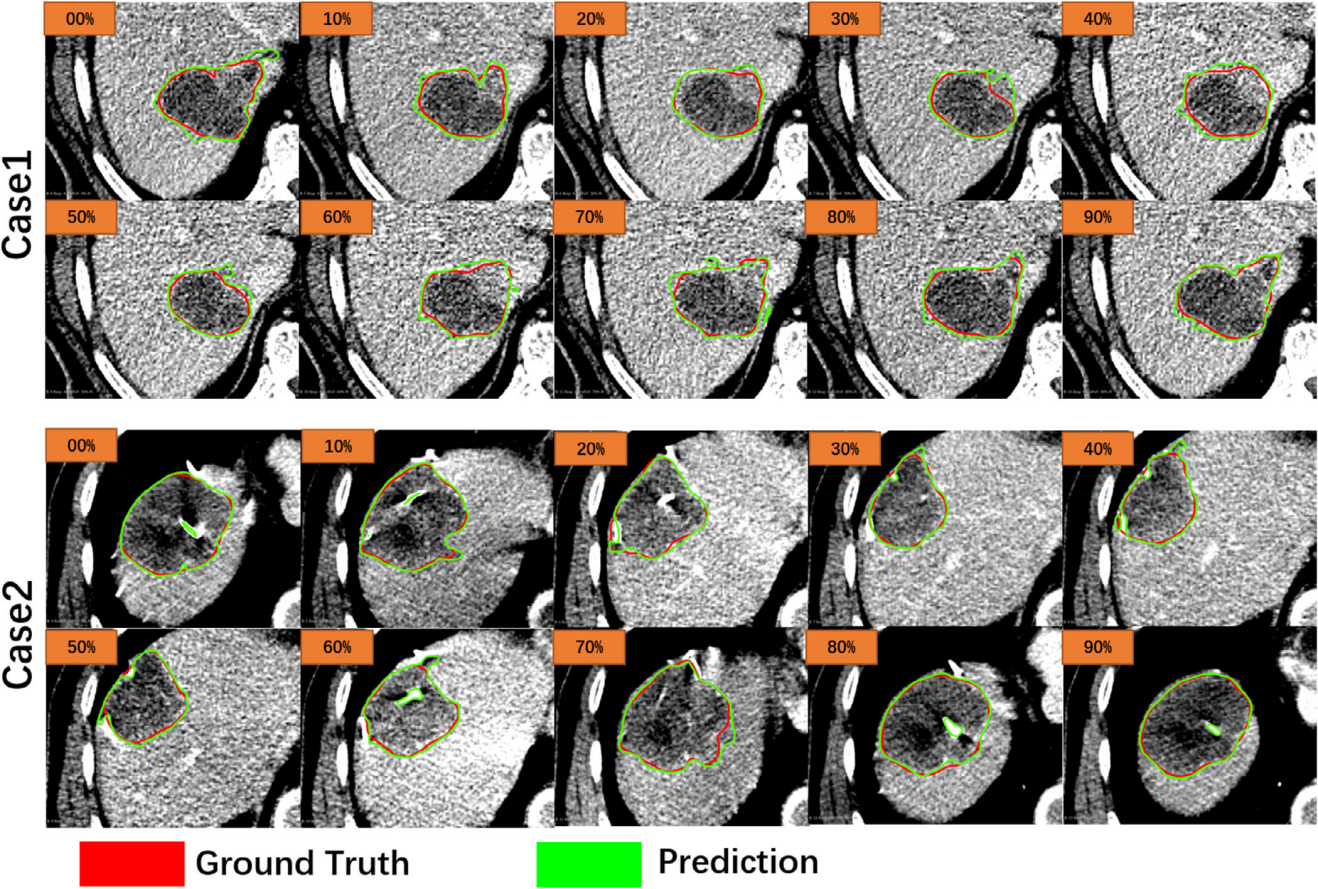
Visualization of GTV segmentation results. The green line is the automatic segmentation result, and the red line is the manual segmentation result by the radiation oncologists. 00%−90% correspond to each respiratory phase of 4D‐CT, respectively. The selected slices are at the same level in the superior and inferior directions, and the axial position was randomly chosen.

Figure 6 presents a visual comparison of the automatically delineated GTV boundaries on 3D‐CTs corresponding to each respiratory phase of two patients. The red labels are the ground truth, and the green labels are the automatic segmentation results. Here we can see that the proposed model can accurately locate GTV regions and precisely segment their contours. In some CT slices, however, there exists over‐segmentation, and the model cannot accurately identify high‐intensity regions within the ground truth regions as predicted GTV. Generally speaking, though, the results of our proposed method agree with the ground truth. The visible tumors in CTs can be precisely predicted, and the demarcation between tumors and normal tissue and low‐intensity regions can be accurately identified. Since the IGTV contains all GTV contours, the under‐segmented GTV on some slices is compensated for by GTV on other slices, which results in closer agreement of the predicted IGTV with the ground truth and a higher DSC score.

**FIGURE 7:**
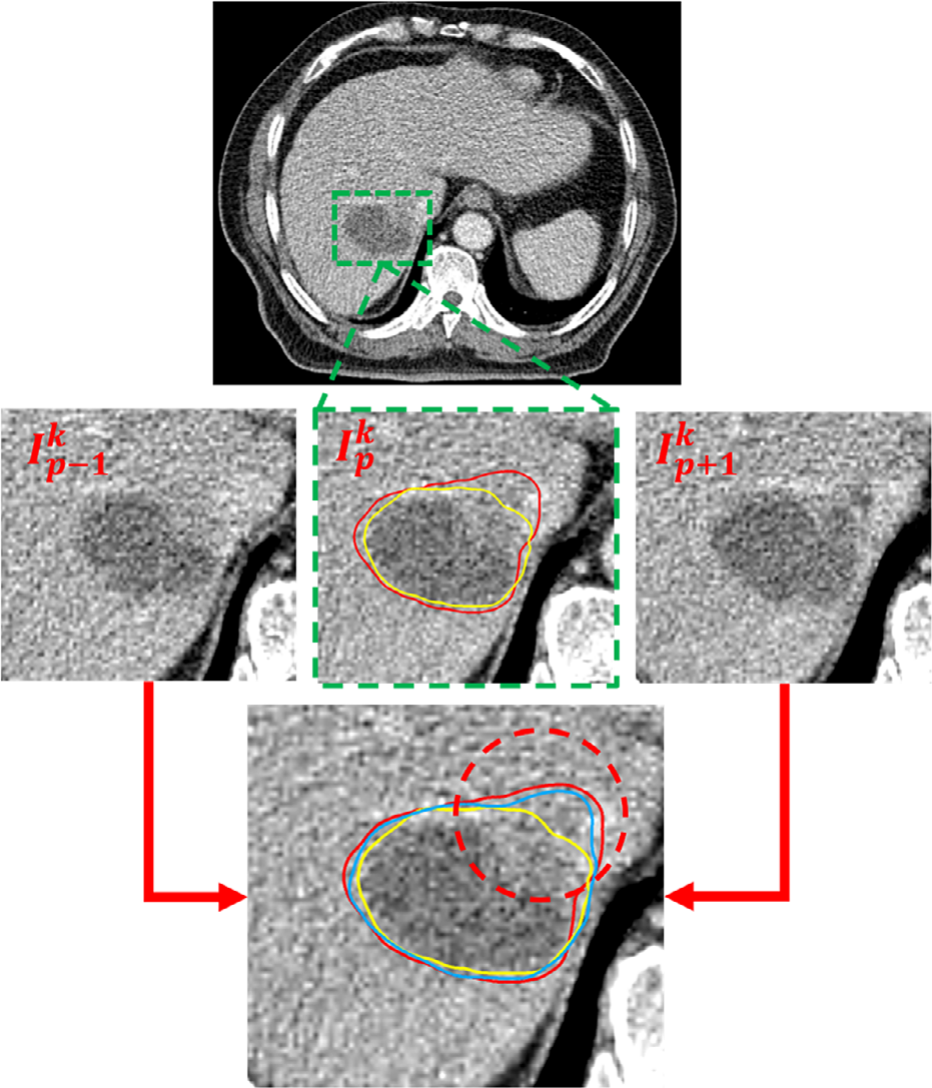
The effect of temporal features on segmentation performance is obvious. The introduction of features extracted from adjacent temporal phases can improve the accuracy of classifying fuzzy regions. The yellow line is the result of U‐Net, the blue line is the result of proposed method, and the red line is the ground truth.

Figure 7 demonstrates the effects of fusing temporal features to enhance the details of GTV segmentation, with the ground truth labeled in red, the results of U‐Net labeled in yellow, and the results of the proposed model labeled in blue. The motion of the tumor is often presented in the image as a blurred boundary, which is very different from both the hypodensity presented by the tumor and the hypodensity presented by the surrounding normal liver tissue, as shown in the red dashed area in the figure. Statistical evidence indicates that the average HU value of such regions is closer to that of the normal liver in most cases. In clinical practice, radiation oncologists empirically classify such regions as GTV considering the motion of tumors, but it is difficult for DL models to make this classification correctly. The introduction of temporal features can solve this problem, however. $I_{p-1}^{k}$ and $I_{p+1}^{k}$ in the figure are the previous and the following temporal phase of the center image, respectively, and the image from $I_{p+1}^{k}$ clearly shows that this region should be included in the GTV range. This indicates that the proposed model can learn the tumor's motion and accurately predict the spatial details as a GTV region by extracting temporal features.

## 4 DISCUSSION

The challenges of clinical GTV delineation of HCC twofold. First, the delineated GTV cannot account for the actual range of motion of the tumor if only static imaging data is used, which may result in the target volume being either too large or too small, leading to unnecessary irradiation of normal tissue or target under coverage. Second, liver tumors are not clearly visualized on regular CT due to insufficient contrast with surrounding healthy liver tissue, making it difficult to delineate them directly and accurately. 4D‐CT‐based GTV delineation has been the state of the art for radiotherapy of HCC, but it has high labor and time costs. To alleviate the tremendous workload of manually delineating GTV contours by radiation oncologists, we proposed a DL‐based approach to automatically detect and segment the GTVs in all respiratory phases in 4D‐CT datasets. Our proposed dual‐path network is based on the U‐Net framework, and our choice to do so was based on the following concerns. First, the semantic information of 4D‐CT images at each respiratory phase is simple and has a fixed structure, so there is no need for screening and filtering too much extraneous information. Moreover, all the images' low‐level and high‐level semantic features are essential, so the U‐shaped structure's skip connection (feature concatenation) can be more effective than other methods. Second, there are so few 4D‐CT images of HCC available for training that it is easy to overfit using a large network such as a DeepLabv3+ model. However, we would like to state that U‐Net models are not designed to model temporal dependencies between time steps in a time series; they do not have a memory mechanism, so they may not be well suited for tasks that require capturing long‐term patterns in the data. We believe that adding the ConvLSTM module to the temporal path may improve the model's accuracy.

Although some competitions for liver tumor segmentation based on regular 3D CT are in progress, they are largely aimed at testing the performance of segmentation algorithms. We believe that it is not feasible to delineate IGTV directly and automatically based on static 3D‐CT because the HU values of tumors and surrounding liver tissues in some slices on regular CT scans are very close to each other, with relatively low contrast that visually presents as fuzzy tumor boundaries and causes a DL network to be easily overfitted during training.

We measured the HU values of the GTV region and the surrounding normal liver tissue (liver minus GTV) at the same horizontal plane of 4D‐CTs and 3D‐CTs from the same patients. The statistical results are presented in Table 5, where we can see that the HU values of the two regions are incredibly close to each other, and it is not easy to distinguish the difference between them with the naked eye. 4D‐CT and regular 3D‐CT of the same patient were scanned sequentially and separately, so contrast weakening may be partly responsible for the low contrast of 3D‐CT. However, there is no need to delineate the tumor on 3D‐CT with contrast, as it cannot capture the motion of the organ.

**TABLE 5:** Comparisons of HU values on GTV and Liver minus GTV.

	Hounsfield Unit (HU)			
Type of CT scan		Minimum	Maximum	Mean
4D‐CT	GTV	−171	497	25.0
	Liver minus GTV	27	228	106.5
Regular 3D‐CT	GTV	−4	470	52.1
	Liver minus GTV	10	92	53.3

The following issues also need to be considered. First, while this work aimed to evaluate our method's performance on a clinical 4D‐CT dataset, our method could be compromised if the CT images themselves contain artifacts introduced by irregular respiratory motion during the scan. Second, there is a large amount of data involved for each patient because DL networks need to be fed with enough data for training. A 4D‐CT typically consists of 10 respiratory phases, each with a different GTV label, involving a much more significant amount of image data than a regular 3D‐CT. In this preliminary study, we trained our network using only 18 patients' data, sufficient to obtain a high segmentation accuracy. Our test dataset used another 5 patients' data and obtained an average DSC of 0.868 for GTV segmentation in all phases. Then we merged all predicted GTVs to obtain predicted IGTVs, compared them with the ground truth, and obtained a DSC of 0.882. Since we used in‐house data from our institution and no comparable studies based on 4D‐CT segmentation of liver tumors were available, algorithmic comparisons could not be performed. As a reference, we reviewed the open leaderboard of the LITS challenge, where the top‐ranked algorithm for liver tumor segmentation obtained an average DSC of 0.825. To delineate the tumors and tumor bed areas accurately on 4D‐CT, radiation oncologists may place metallic markers inside the tumors, which present high intensities in CT images. However, it is evident from the visual presentation of the automatic delineation results that this noise has a limited impact on the network's training and does not profoundly affect the accuracy of the results. Our model is also sensitive to the boundaries of the low‐intensity regions outside the liver and can accurately identify these boundaries. This work is not pioneering in terms of DL‐based methods to segment organs. However, the automatic 4D‐CT‐based segmentation of HCC IGTV is a new effort with positive implications for medical physics (especially for radiation oncologists and physicists).

## 5 CONCLUSION

In this paper, we proposed a dual‐path network that considers spatial‐temporal features, designed a feature fusion module, and discussed the feasibility of automatically delineating IGTV based on 4D‐CT. In addition, we tested transfer learning method to segment the liver of 4D‐CT dataset and obtained promising results. Our proposed method can not only solve the difficulty of accurate automatic delineation on 4D‐CT but can also exploit the fact that on 4D‐CT, tumors have higher contrast and more distinct boundaries with their surrounding tissue, which leads to more accurate automatic delineation. The results of this study demonstrate the effectiveness of our method, which we believe can greatly improve the workflow of 4D‐CT treatment planning and as a result greatly improve patient outcomes.

## AUTHOR CONTRIBUTIONS

**Zhen Yang** and **Shuzhou Li**: Conceptualization; methodology; software. **Xiaoyu Yang** and **Shuanhu Di**: Data curation; writing— original draft preparation. **Ying Cao**: Visualization; investigation. **Qigang Shao**: Supervision. **Du Tang**: Software; validation. **Zhao Peng** and **Yuqian Zhao**: Writing— reviewing and editing.

## CONFLICT OF INTEREST STATEMENT

The authors declare no conflict of interest.

## Data Availability

The data that support the findings of this study are available on request from the corresponding author. The data are not publicly available due to privacy or ethical restrictions.
